# Nicotine Acutely Enhances Reinforcement from Non-Drug Rewards in Humans

**DOI:** 10.3389/fpsyt.2017.00065

**Published:** 2017-05-01

**Authors:** Kenneth A. Perkins, Joshua L. Karelitz, Margaret C. Boldry

**Affiliations:** ^1^Western Psychiatric Institute and Clinic, University of Pittsburgh School of Medicine, Pittsburgh, PA, USA

**Keywords:** nicotine, reinforcement enhancement, reward, smoking, abstinence consequences

## Abstract

Preclinical research documents that, aside from the primary and secondary reinforcing effects of nicotine intake itself, nicotine also acutely enhances the reinforcing efficacy of non-drug reinforcers (“rewards”). Study of these effects in humans has largely been overlooked, but very recent findings suggest they may have clinical implications for more fully understanding the persistence of tobacco dependence. This overview first outlines the topic and notes some recent human studies indirectly addressing nicotine effects on related responses (e.g., subjective ratings), explaining why those findings do not *directly* confirm enhancement of behavioral reinforcement *per se* due to nicotine. Then, the methodology used in the subsequently presented studies is described, demonstrating how those studies specifically did demonstrate enhancement of reinforced responding for non-drug rewards. The main section focuses on the limited controlled research to date directly assessing nicotine’s acute reinforcement-enhancing effects in humans, particularly as it relates to reinforced behavioral responding for non-drug rewards in non-human animal models. After detailing those few existing human studies, we address potential consequences of these effects for dependence and tobacco cessation efforts and then suggest directions for future research. This research indicates that nicotine *per se* increases responding in humans that is reinforced by some rewards (auditory stimuli *via* music, visual stimuli *via* video), but perhaps not by others (e.g., money). These reinforcement-enhancing effects in smokers are not due to dependence or withdrawal relief and can be restored by a small amount of nicotine (similar to a smoking lapse), including from e-cigarettes, a non-tobacco nicotine product. Future clinical research should examine factors determining which types of rewards are (or are not) enhanced by nicotine, consequences of the loss of these nicotine effects after quitting smoking, potential individual differences in these effects, and the possibility that nicotine *via* nicotine replacement therapy and non-nicotine quit medications may attenuate loss of these effects upon quitting. Further study with humans of nicotine’s reinforcement-enhancing effects may provide a more complete understanding of smoking persistence and added mechanisms of cessation medication efficacy.

## Introduction

The notion that nicotine intake critically reinforces tobacco smoking behavior was not widely accepted until the 1980s (1). In subsequent research, nicotine was recognized as having clear primary reinforcing effects, in that non-humans (2–4), and later, humans (5, 6) were shown to self-administer nicotine *per se*. Nicotine also has been found to have secondary reinforcing effects, as environmental stimuli commonly associated with nicotine intake (e.g., discriminative stimuli for nicotine, or “cues”) come to reliably increase behavior (7, 8). These primary and secondary reinforcing effects of nicotine have been the focus of the vast majority of research on nicotine’s acute influences on reinforced behavior (9–11).

However, more recent research, conducted almost solely in non-human animal models, has documented a third reinforcing effect of nicotine on behavior that of enhancing reinforcement from some rewards *not* directly associated at all with nicotine intake (12–16). Here, “reward” will be used to refer to stimuli made available contingent on a behavioral response that increase the subsequent rate of that responding [and thus are apparently reinforcing (17)]. This preclinical research has clearly shown that nicotine *per se* increases behavioral responding that is reinforced by a variety of non-drug stimuli already determined to be rewards (e.g., lights and tones), independent of nicotine’s simple psychomotor stimulant effects. As comprehensively reviewed by Caggiula et al. (18) and Rupprecht et al. (19), nicotine administered acutely or continuously, whether in contingent (i.e., self-administered) or non-contingent fashion, produces reinforcement-enhancing effects. Showing these effects in initially nicotine-naïve animals rules out nicotine dependence as a prerequisite. Furthermore, this immediate onset of nicotine’s reinforcement-enhancing effects and their generalizability across different methods of drug administration both sharply contrast with factors necessary for nicotine’s primary reinforcing effects, such as the critical importance of repeated self-administration training sessions involving contingent responding that produces acute drug exposure (18). Somewhat similarly, other research points to different neuropharmacological mechanisms that may be responsible for the reinforcement enhancing, versus primary reinforcing, effects of nicotine (20, 21).

Contrary to this rapidly growing interest in preclinical research on reinforcement-enhancing effects of nicotine (and other drugs) over the past 15 years, these effects in humans have been largely ignored. Because of the influence nicotine’s reinforcement-enhancing effects could have on a fuller understanding of the persistence of tobacco smoking behavior, and perhaps on use of other nicotine products, this near absence of human research attention is surprising. To our knowledge, only several published studies from our lab have *directly* demonstrated the acute reinforcement-enhancing effects of nicotine intake *per se* in humans in a manner closely related to the preclinical findings, *via* controlled tests of behavioral responding that is immediately reinforced by non-drug rewards. (In studies briefly noted in the next section, other types of measures with humans may possibly be relevant, such as self-reported pleasure from non-drug activities being increased by acute nicotine or decreased after acute abstinence. However, those studies will not be described in detail because most failed to assess behavioral responding reinforced by a non-drug reward, rendering them only indirectly related to nicotine’s reinforcement-enhancing effects.)

Therefore, in an effort to increase awareness of this additional reinforcing effect of nicotine in humans, the goals of this overview are to (1) describe the only controlled research to date specifically designed to assess nicotine’s acute reinforcement-enhancing effects in humans and (2) outline potential clinical implications these effects may have on the persistence of dependence and on efforts to quit tobacco smoking. After briefly commenting on other recent human studies that may be supportive, we summarize the methodology and procedures used in the subsequently presented studies intended to directly test the notion that nicotine acutely enhances behavior reinforced by non-drug rewards. We then describe the results from each of those four studies with that procedure programmatically examining some conditions under which these reinforcement-enhancing effects occur in humans. We also address possible ameliorating effects of cessation medications and other drugs in attenuating loss of nicotine’s reinforcement-enhancing actions when quitting smoking, which may identify additional mechanisms by which these treatments could help maintain tobacco abstinence. Extensive discussion of the preclinical findings, including mechanisms of nicotine that may be responsible, is beyond the scope of this paper and has been provided elsewhere (18, 19, 21). Finally, directions for future research into characterizing and understanding nicotine’s reinforcement-enhancing effects in humans are suggested, often drawing on the potentially relevant preclinical results. The intent of this article is to provide the first review of the limited controlled research to date demonstrating nicotine’s acute reinforcement-enhancing effects in humans and to encourage further study of this overlooked phenomenon to determine its potential clinical implications for explaining tobacco use persistence.

### Prior Human Studies of Nicotine Effects on Non-Drug Reward-Related Responses

As noted above, very few studies have directly examined nicotine’s reinforcement-enhancing effects in humans, although other research has assessed various types of responding due to recent nicotine exposure that may relate to those effects. We describe some of that research here, to better define what is intended by the term “reinforcement enhancement” as addressed in the detailed studies comprising the main section of this review. (Those studies follow the description of methodology used in those studies to document such enhancement in humans.) For examples, acute nicotine *via* smoking increased subjective ratings of the attractiveness of pictorial faces (22) or of positive mood during a mood induction procedure in depression-prone smokers (23), and nicotine *via* patch increased correct responding on a challenging signal detection task reinforced by money in non-smokers (24). Further, nicotine *via* lozenge improved performance on a card-sorting task where speed of responding was reinforced by money, but it did so only in heavy and not light smokers (25), suggesting that nicotine-dependent participants were necessary. Comparable research found no effect of acute nicotine *via* “inhaler” on patterns of gambling behavior for monetary reinforcement (26). Because of their indirect or insensitive measures of behavioral reinforcement of non-drug rewards, these and related studies may be quite limited in what they can inform us about the existence of reinforcement-enhancing effects of acute nicotine intake in humans as demonstrated in the preclinical research. These studies also typically failed to assess responding for non-reward stimuli to verify specificity of nicotine’s effects on reinforced behavior *per se* rather than on non-specific responding (17).

Still other research has examined changes in similar types of responses as a function of smoking abstinence, instead of assessing acute effects of nicotine intake. This work generally proposes a disruption in incentive motivation or reward responsiveness due to nicotine abstinence in dependent smokers (27), rather than enhancement of reinforced behavior for non-drug rewards by acute nicotine administration. In one such study, overnight abstinence decreased self-reported pleasure from viewing positively valenced movie clips, relative to non-abstinent smokers and independent of withdrawal (28). On the other hand, smoking abstinence for one or more weeks in those attempting to quit permanently increased, rather than decreased, self-reported enjoyment of various “rewarding events” (29), opposite of that expected by the loss of nicotine’s reinforcement-enhancing effects. Thus, some of this research may relate more closely to withdrawal and anhedonic effects of recent abstinence (30, 31) or could reflect broader and more gradual improvements in overall well-being with successful abstinence (32), rather than loss of nicotine’s acute enhancement of responding reinforced by non-drug rewards.

Due to the reliable preclinical findings on nicotine’s reinforcement-enhancing effects (18, 19, 21), and the lack of prior human research directly examining these effects on reinforced behavior under carefully controlled conditions, we developed a procedure to assess the reinforcement-enhancing effects of acute nicotine on responding for non-drug rewards in humans. Our early piloting indicated a need for altering some methodological details (33) to finalize a procedure that was sensitive to changes in the reinforcing efficacy of some non-drug rewards after acute nicotine intake. Because most aspects of that procedure are common to the clinical studies to be described below, details of that procedure will be presented next, followed by results from the only published research examining factors moderating the presence or magnitude of nicotine’s reinforcement-enhancing effects in humans. (All are from our lab; we know of no other controlled studies specifically designed to assess these acute nicotine effects *per se* on reinforced responding for non-drug rewards in humans.) Conclusions of the human research, potential clinical implications of these reinforcement-enhancing effects for maintaining dependence and impeding success of tobacco cessation, and suggested future research directions are then addressed.

## Procedure to Assess Reinforcement-Enhancing Effects in Humans

### Procedure Rationale and Basic Study Design

Our starting point was to develop and evaluate a testing procedure that matched those from rodent studies as closely as possible. We did so to minimize chances that failure to find reinforcement-enhancing effects of nicotine was due to procedural differences rather than lack of generalizability from non-human animals to humans. For example, to carefully control nicotine administration to participants *via* tobacco, their smoking exposure is determined by computerized instructions on the number, timing, and duration of all puffing behavior, confirmed by puff topography assessment after each administration. Second, our primary dependent measure is responding on a simple button-pressing task (to simulate lever pressing) that is reinforced by immediate delivery of brief rewards under a progressive-ratio (PR) schedule, which minimizes the odds of reward satiation. As the label implies, PR schedules require a progressively greater number of responses for each succeeding delivery of a reinforcing stimulus (i.e., a “unit” of reward) within a session, and the point at which such responding stops is taken as identifying the maximal reinforcing efficacy of that reward (17). Although occasionally used in human studies of reinforced responding for rewards (34), PR schedules are far more common in non-human animal studies (35, 36).

Also, to maximize statistical power (37) and assessment efficiency, testing involves a fully within-subjects design, in which drug condition (i.e., nicotine dose) is manipulated between sessions and the immediate reward condition varies across trials within sessions. Specifically, varying drug condition across three sessions allows us to assess the independent effects of acute nicotine *per se* (e.g., nicotine versus denicotinized cigarette) and of simply engaging in smoking behavior *per se* (denicotinized cigarette versus no smoking). Each condition is provided in counter-balanced order, and the order of reward condition is the same for a given participant across sessions so that only the session’s drug condition is different. (No order effects have been observed for either the drug condition across sessions or the reward condition within sessions.) All sessions start following overnight abstinence (confirmed by expired-air carbon monoxide of CO ≤ 10 ppm) so that subsequent testing of nicotine effects is based on controlled acute exposure due to study manipulations.

### Reinforced Behavior Task

Reinforced responding for non-drug rewards using the PR schedule of reinforcement is assessed with a modified version of a simple computer task developed as a research tool for human studies of reinforcement schedules [“Applepicker” (38)]. It was previously used by us to assess acute reinforcement by access to smoking (i.e., nicotine’s primary reinforcing effects), food or money, or alcohol (39–41). To obtain rewards from using this task, subjects move a keyboard’s cursor around a video monitor to look for “apples” by pressing a button whenever the cursor lands on one of the small filled circles displayed in a grid, symbolizing an orchard of “trees.” The number of button presses (responses) required to find an apple is controlled by the computer’s programmed schedule of reinforcement. Participants themselves determine the duration of their responding, discontinuing only when they decide the response requirement for receiving the next unit of that reward exceeds what they are willing to complete. Total responding within a trial up to that point is used as the measure of that reward’s reinforcing efficacy; because participants will often continue responding after earning one unit of reward but stop prior to earning the next unit of reward, the “break point,” or final completed response requirement, alone may not fully capture the efficacy of that reward for a participant unless the additional responding is also counted. (This schedule was a PR30% in our first study, increased to PR50% in all subsequent studies, meaning each successive reward delivery requires 50% more responses on the task than did the preceding response requirement; e.g., 10 responses to earn reward the first time, then 15, 22, 33, 50, 75, and so on. The PR50% ensures a point of maximum efficacy for that reward is reached before the end of each 15 min trial.) A separate “no reward” trial, in which responding does not result in delivery of any stimulus, is routinely included in our testing sessions to allow assessment of non-specific responding and rule out nicotine’s general psychomotor stimulant effects on behavior. Moreover, responding on the “no reward” trial gauges whatever reward value is provided to the participant by simply engaging in the task [similar to the inactive lever in preclinical research (18)]. The monitor’s graphics are kept rudimentary to avoid the possibility that task responding might provide its own entertainment value in the absence of an available designated reward (i.e., serve independently as a reinforcing stimulus). Also, so that subjects do not simply continue responding on the task simply out of boredom, intentionally routine reading material (that would not be a competing reinforcer) is freely available until the trial’s end.

### Reward Delivery

Finding an apple (i.e., completing a response requirement) earns an immediate reward delivery, or one unit of the designated reinforcer, as with onset of reward stimuli in the preclinical research (18). Each reward is available singly on separate test trials, which are presented in counter-balanced order within sessions across subjects. Thus far, the types of rewards enhanced by nicotine in preclinical studies appear qualitatively distinct (42) but may be characterized as stimuli that are “sensory” in nature [such as visual lights, auditory tones, olfactory, or gustatory tastes (19)]. Therefore, rewards in our human studies now usually include music (auditory), video (visual), and, for comparison, one that is not sensory (money) along with the no reward trial to gauge non-specific responding. Typically, units of reward for completing each reinforced response requirement are 30 s of preferred music or video (positive sensory reinforcement), $0.10 for money (positive non-sensory reinforcement), or no stimulus (no reward control). These music, video, and money positive reward units were selected based on piloting that demonstrated equal reinforcing efficacy between each under controlled conditions. Auditory stimuli are presented through headphones, video is shown in a separate viewing portion of the monitor, and money earned is displayed on a counter shown on the monitor.

Critically, the specific preferred music and video rewards are identified separately by participants during an introductory session by listening to their own supplied musical tracks, or by viewing identified video clips (*via* electronic means), and rating each on a 0–100 visual-analog “liking” scale, with those rated >75 considered “highly preferred.” This procedure of personalizing the music and video rewards ensures each will be clearly reinforcing for that individual, rather than assuming fixed music and video rewards will be comparably reinforcing for all participants (43).

## Assessment of Nicotine’s Reinforcement-Enhancing Effects in Humans

**Figure 1:**
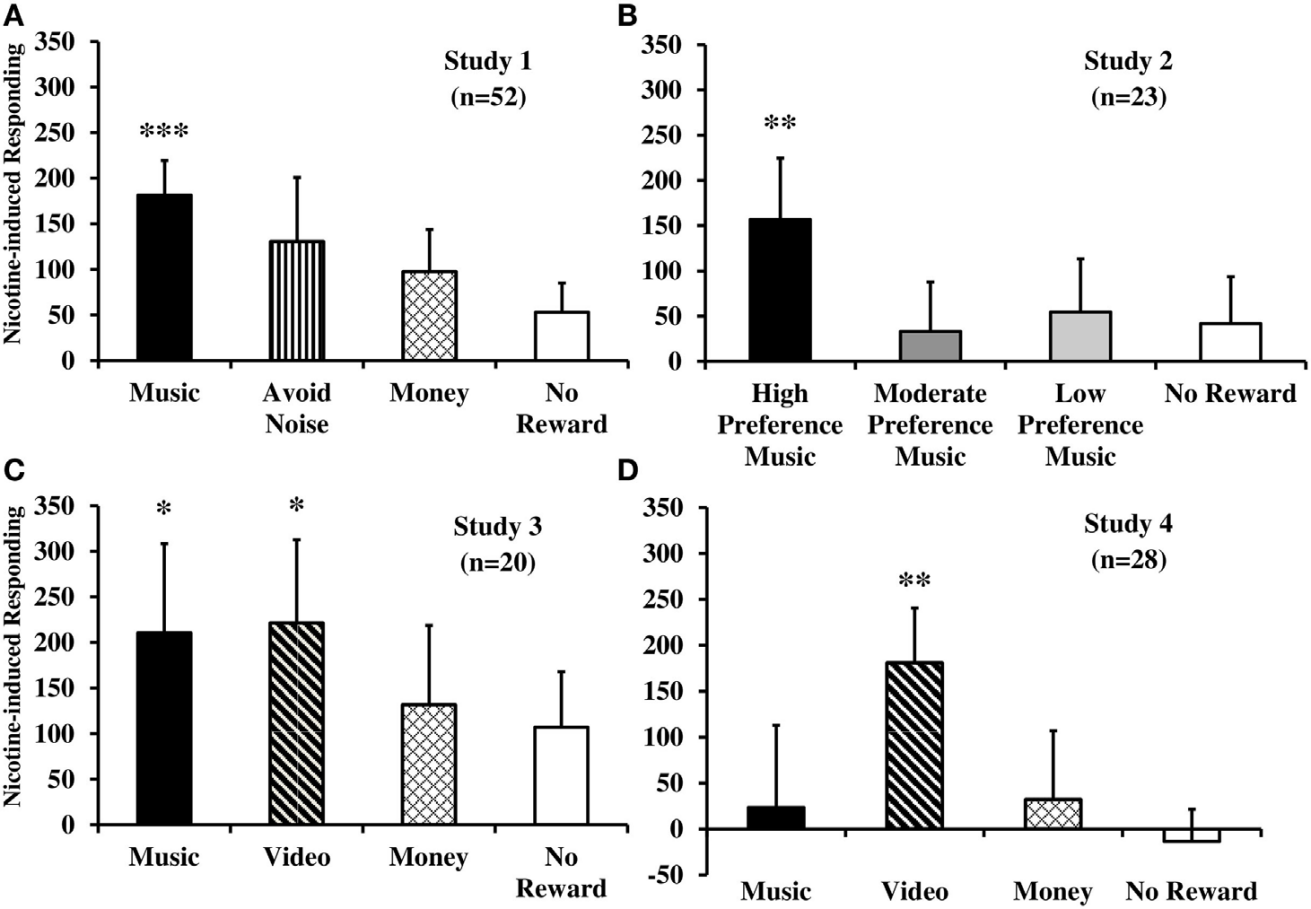
**Nicotine-induced increases in behavioral responding that was reinforced by immediate delivery of non-drug rewards or no reward, separately, for each of four within-subject studies**. Displayed are mean (SEM) total responses due to acute nicotine administration *per se* from tobacco smoking [studies 1–3 **(A–C)**] or e-cigarette [study 4 **(D)**], after controlling for responding due to denicotinized smoking or placebo e-cigarette (***p < 0.001; **p ≤ 0.01; *p < 0.05 for significance of nicotine-induced responding for reward). Responses on the no smoking (studies 1–3) or no e-cigarette (study 4) sessions are not shown, as no effects were ever observed between use of the denicotinized or placebo products versus no use. See text for details of each study.

Results of the total nicotine-induced responding reinforced by non-drug rewards or no reward, after controlling for non-nicotine testing factors, are displayed in Figure 1, separately by each of four studies to be described below. As far as we are aware, these are the only published studies in humans designed to assess in *a priori* fashion the effects of nicotine *per se* on enhancement of reinforcement from non-drug rewards, while controlling for the non-nicotine effects of testing (i.e., smoking behavior *per se*) as well as addressing possible non-specific effects on responding.

### Enhanced Reinforcement in Dependent and Non-Dependent Smokers

We first used this procedure to examine the influence of nicotine (versus denicotinized) cigarettes on enhancing positive reinforcing effects of the sensory and non-sensory rewards of music and money, respectively, in 52 smokers (44). To further explore generalizability, we also assessed responding for the negative reinforcing effect of avoiding intermittent aversive white noise (for 30 s per reward unit) as well as for the no reward control of non-specific behavior. As noted above in the description of basic design procedures common across studies, participants were administered three different smoking conditions on three separate experimental sessions, each following overnight abstinence, to compare responses due to no smoking with those due to administration of 0.05 or 0.6 mg nicotine Quest brand cigarettes under blind conditions. Six puffs on either cigarette over 3 min preceded each trial on the two smoking sessions. Finally, our first study also confirmed that these effects do not require the presence of nicotine dependence or withdrawal, as in the preclinical research (18). To do so, we compared reinforced responding for non-drug reward due to nicotine in 25 dependent versus 27 non-dependent smokers, who averaged 14.3 versus 1.5 cigs/day, respectively.

Reinforced responding for music, but not money or avoidance of aversive noise (or no reward control), was significantly increased by the 0.6 versus 0.05 mg nicotine cigarette (see Figure 1A), confirming reinforcement-enhancing effects of nicotine intake *per se* on a positive sensory reward in humans. No differences in responding between smoking the 0.05 mg cigarette versus the no smoking condition indicated no effects of simple smoking behavior *per se* (without much nicotine) on any rewards. These findings due to nicotine were consistent between the dependent and non-dependent smokers, as no between-group differences in reinforced responding were found. Similarly, responding due to this smoking was unaffected by withdrawal relief, as elevated withdrawal at baseline in dependent smokers declined post-nicotine to that of non-dependent smokers, whose withdrawal score was low at baseline and remained low throughout sessions (44). Thus, there was no difference in reinforcement-enhancing effects of nicotine as a function of being a dependent or non-dependent smoker or due to nicotine’s actions in withdrawal relief. We believe this was the first direct demonstration of the reinforcement-enhancing effects of acute nicotine administration in humans.

### Magnitudes of Nicotine Exposure and of Reinforcer Efficacy Needed for Effects

The next step was to examine whether nicotine’s acute effects on enhancing reinforcement from music may vary due to the amount of acute smoke intake (i.e., nicotine dose), and the available reinforcer’s magnitude of efficacy (45, 46). Using a within-subjects design and procedures similar to our first study, 23 dependent smokers in study 2 completed 3 experimental sessions after overnight abstinence (47). The three sessions were virtually identical, differing only in the modest number of puffs from their preferred nicotine cigarette brand (i.e., unblinded) prior to testing: (1) eight puffs pretesting and then two puffs per trial, (2) two puffs per trial only, or (3) no smoking exposure at all. Each session involved separate trials of responding for reinforcement from high (rated >75 on 0–100 scale), moderate (rated 40–60), or low (rated 0–20) preference music rewards (or no reward control). As in study 1, rewards were personalized and identified separately by each participant during an introductory session, given individual differences in music preferences (43).

As expected based on their identified preferences, overall responding for reinforcement was influenced by the self-reported preference level of music reward. More importantly, responding for the high preference music trial, but not the moderate or low preference music or no reward trials, was increased after smoking eight puffs pretesting plus two per trial, compared to the two per trial only or no smoking sessions, which did not differ (Figure 1B). Again, withdrawal did not differ between the two smoking sessions, ruling out withdrawal relief due to nicotine as an explanation for the different responses for the high preference music reinforcement. These results confirmed that just over eight puffs from one cigarette after abstinence (as in a “lapse”) may be enough nicotine to obtain its reinforcement-enhancing effects, while very minimal smoking of just a few puffs is not. Findings of study 2 further showed the specificity of nicotine’s effects by its enhancement of responding for music reward that was high preference but not music of lesser preference (i.e., lower magnitudes of reinforcing efficacy) or for the no reward control (47).

### Generalizability of Nicotine’s Enhancing Effects to Other Sensory Rewards

Although the reinforcement-enhancing effect of nicotine on music reward in humans in study 1 was replicated in study 2, it was important to document its generalizability to other common sensory rewards in a smoker’s environment, in addition to music. Given preclinical research finding nicotine’s enhancement of responding for visual rewards (18), we chose to examine whether a visual reward would be enhanced by nicotine in humans similar to that observed with the auditory reward of music. Thus, in study 3 (48), we assessed acute effects of nicotine *via* smoking on responding for music and for video rewards, along with the same monetary reward or no reward control comparison conditions as in study 1. Preferred video rewards were identified for each individual in the same way as preferred music, as described previously. Also similar to study 1, a fully within-subjects design was used in study 3, in which 20 dependent smokers participated in three experimental sessions following overnight abstinence, differing only in the smoking conditions of six puffs on a 0.6 or 0.05 mg nicotine cigarette under blind conditions, or no smoking, prior to each task trial.

As hypothesized, reinforced responding for music and video rewards, but not for money (or no reward), was greater due to smoking the 0.6 versus 0.05 mg cigarette (Figure 1C), showing effects of nicotine *per se* (48). Lack of differences between the 0.05 mg cigarette and no smoking showed no effects of smoking behavior *per se* on reinforcement from music or video reward. Once again, effects were not influenced by withdrawal relief from either cigarette. Study 3’s results, extending nicotine’s reinforcement-enhancing effects from an auditory (music) to a visual (video) reward, were generally consistent with those from the preclinical research briefly noted earlier (19). They confirmed that acute nicotine intake *per se* enhances the reinforcing value of multiple sensory rewards (42).

### Non-Smoked (e-Cigarette) Nicotine Effects on Enhancing Reinforcement

An important factor needed to more closely link the human research to preclinical findings was to demonstrate that nicotine *via* a non-smoked manner of intake would also have reinforcement-enhancing effects. Studies 1 and 3 had manipulated nicotine exposure by administering cigarettes differing in nicotine delivery under blind conditions, so that all the non-nicotine factors accompanying nicotine intake would be controlled. To examine nicotine effects in the absence of tobacco smoke, we repeated the fully within-subjects design and procedures from study 3 (48), described above, but using electronic (e-) cigarettes differing in nicotine content (under blind conditions), rather than tobacco cigarettes. Thus, on three sessions after overnight abstinence, 28 dependent smokers in study 4 were administered an e-cigarette with vapor containing nicotine (36 mg/ml), one containing no nicotine, or no e-cigarette at all, prior to each trial in which they responded for the music or video sensory rewards, monetary reward, or no reward (49). Comparable to dosing in the smoking studies above, use of e-cigarettes was carefully controlled by precise instructions on timing and duration of 10 puffs over 5 min, a procedure shown to deliver rapid rises in blood nicotine (50).

Mostly similar to our prior studies of nicotine *via* tobacco smoking, reinforced responding for the video reward, but not for the other rewards (including music), was greater after the nicotine versus placebo e-cigarette (Figure 1D), confirming some reinforcement-enhancing effects of nicotine *per se* administered in a non-smoked formulation (49). Also as in study 3, no differences were seen due to behavioral effects of e-cigarette use *per se* without nicotine (i.e., responding due to the placebo e-cigarette in comparison with the no e-cigarette session). Replication of study 4 is needed to verify that music reward may not be enhanced by this amount and manner of nicotine intake from e-cigarette use despite very reliable music reward enhancement from nicotine *via* tobacco smoking (see studies 1–3 in Figures 1A–C), a result not easily explained. Aside from partly confirming that acute nicotine from a non-tobacco product has some reinforcement-enhancing effects in humans, as with tobacco cigarette smoking, these results could help explain the growing prevalence of nicotine e-cigarette use in the wider population (51).

### Summary of Human Studies

Although much more research is needed, the clinical studies described here confirm the essential preclinical finding that nicotine enhances reinforcing effects of some non-drug rewards unrelated to nicotine. Study 1 indicated that nicotine *per se* acutely increases reinforced responding for music reward but not for money or the negative reinforcer of terminating aversive noise and that these effects occur with non-dependent as well as dependent smokers, as in the non-human research (18). Study 2 showed that nicotine from barely one cigarette acutely enhances responding reinforced by high preference music, but not by lesser preference music or from minimal cigarette smoking. Generalizability of reinforcement-enhancing effects of nicotine across types of sensory rewards, from music to video, in study 3 showed consistency with findings on reinforcing auditory and visual stimuli as rewards in preclinical studies. In study 4, some reinforcement-enhancing effects of nicotine from a non-tobacco, non-smoked method of administration (e-cigarette) was demonstrated for video reward, confirming effects in humans are not specific to nicotine *via* tobacco smoking. In all studies, the behavior of simply puffing on a tobacco or electronic cigarette alone did not alter reinforced responding, and withdrawal relief was unrelated to nicotine’s reinforcement-enhancing effects.

## Potential Clinical Implications

### Smoking Cessation Effects

Given how commonly available many sensory rewards are in everyday life (52), including while smoking in environments where it is not restricted (53), an overlooked factor contributing to smoking’s persistence may be nicotine’s ability to enhance the reinforcing efficacy of these rewards. Recent U.S. population surveys indicate engaging in “leisure” activities containing such rewards may comprise about 5 h/day, or nearly one-third of waking hours (http://www.bls.gov/TUS/CHARTS/LEISURE.HTM). Therefore, an attempt to quit smoking also could lead to a loss of nicotine’s acute reinforcement-enhancing effects when quitters are engaged in non-drug activities providing sensory rewards, as clearly suggested by preclinical research on abrupt discontinuation of nicotine infusions (54).

To begin to understand the loss of nicotine’s reinforcement-enhancing effects that smokers may experience when initially attempting to quit smoking, we recently assessed responding reinforced by these sensory rewards during typical ad lib smoking compared to abstinence, in a two-session within-subjects design (55). Prior to the two sessions, 48 dependent smokers (non-treatment seeking) either abstained overnight (CO < 10 ppm) or smoked their own preferred brand in completely ad lib fashion (i.e., to capture typical nicotine satiation while smoking). During each session, they responded on the same task used in the above studies in three separate trials reinforced by the music or video sensory rewards, or for the no reward control. Relative to the ad lib smoking session, overnight abstinence significantly reduced reinforced responding for both sensory rewards but not for the no reward control condition (55), verifying sharp loss of these nicotine effects early in a quit attempt, similar to preclinical findings (54). As with our controlled studies of acute smoking after overnight abstinence, above, differences between sessions in withdrawal, which were substantial as expected, were not related to the differences between sessions in reinforced responding.

Consequently, quitting smoking may lessen overall reinforcement from these non-drug related rewards, separate and independent of withdrawal symptoms and other consequences of cessation. Although of uncertain impact without more direct clinical study of these changes, a decline in the rewarding effects of these activities could possibly contribute to a greater risk of lapse (and relapse) in an effort to restore this loss in reinforcement. Perhaps consistent, recent clinical research suggests early lapses after starting a quit attempt are common during leisure activities, including while concurrently enjoying “TV/music” (56). In short, aside from nicotine’s primary and secondary reinforcing effects, tobacco smoking may be particularly difficult to quit for long partly because acute nicotine makes engaging in so many of these typical daily activities more pleasurable. Therefore, a sudden lessening of this degree of pleasure from familiar rewarding activities after abstaining from smoking may help prompt a lapse, which then could foster complete relapse (57).

### Medications May Limit Loss of Reinforcement-Enhancing Effects after Quitting

In addition to moderating craving and other withdrawal symptoms of abstinence, as well as blunting nicotine’s primary reinforcing effects, medications to aid smoking cessation could partly act by attenuating loss of nicotine’s reinforcement-enhancing effects after quitting tobacco use. No clinical research has formally addressed this notion, but some human studies are suggestive. For example, our findings above in study 4 showed potential for nicotine *via* e-cigarettes to prevent some of the loss of reinforcement-enhancing effects of nicotine *via* tobacco smoking after abstinence [although e-cigarette use has not been clearly demonstrated to aid smoking cessation (58)]. Other non-smoked nicotine products, especially the various U.S. FDA-approved nicotine replacement therapy (NRT) medications intended to help smokers quit (e.g., patch, lozenge, and gum, inhaler), may also enhance reinforced responding for sensory rewards. If confirmed in clinical research, these actions could further help explain why NRT reduces lapse risk and can prevent smoking lapses from turning into complete relapses (59).

Moreover, *non*-nicotine medications for cessation may also aid quitting partly by attenuating the drop in reinforcement from sensory rewards due to loss of nicotine intake after abstinence. Because of preclinical research showing that reinforced responding for rewarding stimuli was enhanced by bupropion (60), we conducted a small pilot study of this notion with bupropion in smokers recruited due to their high interest in making a permanent quit attempt. All were tested on three separate sessions, first after ad lib smoking and then during a crossover comparison of bupropion versus placebo, each after initiating a brief “practice” quit attempt period. In those meeting 24-h abstinence criteria during both medication conditions (CO < 5 ppm), reinforced responding for the high preference music reward decreased significantly, by almost half, when they quit while on placebo, while their responding after quitting on bupropion was similar to that during the ad lib smoking session (61).

Based on these modest findings, formal testing of NRT and of bupropion in attenuating loss of nicotine’s reinforcement-enhancing effects among quitting smokers may be obvious clinical research to conduct. Another may be to similarly test effects of varenicline, a partial agonist of nicotine that is FDA approved for cessation, on enhancement of reinforced responding. In rodents, moderate-dose varenicline enhances reinforcement from visual stimuli (62, 63), but other research indicates varenicline’s effects are less robust than those of nicotine, perhaps consistent with varenicline’s partial agonist actions (64).

### Other Clinical Research Directions

Also needed are studies to more fully characterize nicotine’s reinforcement-enhancing effects in humans. These include determining other types of rewards that are enhanced by nicotine, potential individual differences in these enhancing effects by nicotine, and the possibility that nicotine may enhance a reward by attenuating habituation to its reinforcing efficacy, all of which will be addressed next. In addition, identifying what other drugs may have reinforcement-enhancing effects in humans could help determine pharmacological mechanisms contributing to these effects, as well as broadening the focus of enhanced reinforcement from non-drug rewards to other substance abuse problems. Preclinical research provides several directions to guide this human research.

#### Identifying Other Rewards Enhanced by Nicotine

Aside from audio and visual stimuli from the music and video rewards enhanced by nicotine in our studies, nicotine may also enhance the reinforcing effects of olfactory/taste stimuli (42, 65, 66) and perhaps social rewards (67). Some of these olfactory/taste stimuli could be sensory rewards unrelated to smoking (68) that are enhanced by nicotine intake, as with music and video. However, other specific sensory stimuli become conditioned reinforcers (cues) from repeated association with nicotine’s primary reinforcing effects *via* tobacco smoking [e.g., taste and smell of smoke inhalation (69, 70)]. Those stimuli could become even more rewarding due to nicotine’s acute reinforcement-enhancing effects, beyond the conditioned reinforcing effects of these stimuli (66). Research confirming similar effects in humans could help explain why the taste and smell of cigarette smoke are such potently rewarding components of the tobacco smoking experience (8, 39, 71), factors that could also influence e-cigarette use and enjoyment (72, 73). Comparable human research examining nicotine’s potential reinforcement-enhancing effects of social rewards may contribute to an understanding of how smoking behavior so typically becomes an activity often done with other smokers (74–76).

Rather than rewards enhanced by nicotine being those “sensory” in nature, a behavioral economic examination may help clarify characteristics of rewards that are or are not likely to be enhanced by nicotine intake. A common behavioral economic approach is to relate how other reinforcers may acutely increase or decrease subsequent drug consumption, in this case tobacco smoking behavior. For example, reinforcers (i.e., rewards) in which increasing their availability is associated with an increase in smoking are labeled “complements.” By comparison, those reinforcers associated with a decrease in smoking are “substitutes,” and those unrelated to smoking are “independent” (77, 78). However, the reverse relationship may also be very relevant, as nicotine intake from smoking can increase consumption of some non-drug rewards (e.g., the music and video rewards from our studies above), and so smoking could complement those rewards. Rewards not affected by nicotine (e.g., money) may be independent, and any in which consumption is decreased by nicotine may be substitutes. It is conceivable, therefore, that rewarding activities and smoking behavior may have mutually reinforcing relationships, as certain activities make smoking more reinforcing and intake of nicotine from smoking makes certain activities more enjoyable. Thus, these activities and smoking may be complementary influences on each other. Perhaps similarly, recent clinical research indicates smoking cessation may be more successful in those who decrease participation in complementary rewarding activities (79), which presumably would lessen ex-smokers’ exposure to loss of reinforcement-enhancing effects due to abstinence from nicotine.

#### Potential for Individual Differences in Nicotine’s Reinforcement-Enhancing Effects

Also relevant is the possibility of individual differences in the magnitude of reinforcement-enhancing effects of nicotine (80), including as a function of the smoker’s typical frequency and pattern of smoking (81). Development of these effects during initiation of smoking behavior in adolescents could inform factors influencing escalation to greater tobacco use and dependence (82). Individual difference characteristics of smokers themselves also could affect the degree or impact of nicotine’s reinforcement-enhancing effects, possibly contributing to differences in the prevalence and persistence of smoking status in those with the characteristics. For example, the majority of adults in the U.S. with any mental illness have a lifetime history of smoking, and they have much lower quit success and are two to four times more likely to be a heavy smoker than those without mental illness (83). Conceivably, then, compared to adults without mental illness, those with mental illness could tend to experience even greater magnitude of reinforcement-enhancing effects from consuming nicotine (e.g., greater subjective pleasure from the non-drug rewarding stimuli). Alternatively, perhaps, these enhancing effects the mentally ill experience from smoking may have more of a mood-regulating benefit for them relative to other smokers, even if the absolute magnitude of enhanced sensory reinforcement *via* nicotine exposure is comparable between these subgroups. Also warranting further study is examination of how these reinforcement-enhancing effects of nicotine may be moderated by the specific testing conditions employed (46), as concurrent environmental situations and experiences by participants (e.g., subjective distress) could further increase, or attenuate, such enhancement by nicotine of responding that is reinforced by non-drug rewards (84, 85).

#### Enhancing Reward by Attenuating Habituation to Its Reinforcing Efficacy

The acute effectiveness of a non-satiating (i.e., not biologically necessary) reward in maintaining responding decreases incrementally with each presentation of the reward, a process termed “habituation.” So, nicotine may also enhance duration of reinforced responding by maintaining the effectiveness of the same reward across repeated presentations during one period of access, countering the systematic decline in responding typically attributed to habituation of a reinforcer’s effectiveness (86). Preclinical research shows that nicotine (and methamphetamine) slows habituation of the reinforcing effectiveness of a visual reward (87). Such an effect may be more pronounced for rewards that are intermediate in reinforcing efficacy between those extremely desired versus minimally desired, which may produce very slow versus very fast habituation, respectively (88). In short, nicotine’s ability to delay habituation of a reinforcer’s effectiveness in preclinical studies may partly help explain how it enhances overall responding for that reward, and this possibility warrants clinical research attention in humans.

#### Other Drugs May Enhance Reinforcement in Humans

Finally, other research with human participants also could explore the degree to which preclinical findings on the reinforcement-enhancing effects of other drugs show cross-species generalization, within practical and ethical limitations. For example, several “stimulant” drugs have also been shown to increase reinforced responding for non-drug rewards in animal models, such as caffeine (89), methamphetamine (90), cocaine (91), phencyclidine (92), and other stimulants (93). These findings may support the notion that specific dopaminergic receptor activity is key to eliciting reinforcement-enhancing effects (21). Additional research in humans may be able to confirm generalizability of neuropharmacological underpinnings of these drug actions, as identified by preclinical research (21, 94). Our procedures in the human studies described above may also be applicable to conducting research on what may be reinforcement-enhancing effects of other drugs in humans (95).

## Conclusion

Despite only sparse clinical research with humans, these findings appear largely consistent with preclinical studies on the reinforcement-enhancing effects of nicotine. Rewards that are enhanced by nicotine may be characterized as positively reinforcing stimuli that are sensory in nature (96), and specificity is strongly indicated in that nicotine intake never enhanced reinforced responding for the non-sensory reward of money in any study. We also found no effect of nicotine on enhancing responding for a no reward control condition, or in one study for a negatively reinforcing stimulus (terminating aversive noise). However, viewing these enhanced rewards as “complements” of smoking behavior, in behavioral economic terms, may characterize them in a way that more broadly informs future research into other potential rewards that could be enhanced by nicotine. Importantly, and consistent with the preclinical research, we found no evidence that withdrawal relief or presence of nicotine dependence are necessary to explain nicotine’s reinforcement-enhancing effects.

Further clinical research on the implications of these effects for smoking persistence and cessation is justified by this initial work in humans, combined with the more extensive preclinical research findings. Confirming that a decrease in the reinforcing value of non-drug sensory rewards occurs after making a permanent attempt to stop smoking would be important, given frequent exposure of smokers to these types of rewards over the course of typical daily living. A rapid decline in enjoyment of these common rewards, as we observed (61), may contribute to lapsing soon after a quit attempt, as modest nicotine intake can be sufficient to restore the reinforcement-enhancing effects. Yet, whether or not a decrease in these effects may interfere with subsequent success in maintaining abstinence remains to be determined. Further demonstration that non-smoked nicotine can also restore these effects may help explain an additional beneficial action of NRT medication for cessation of tobacco. Because bupropion appears to show similar effects, and preclinical findings with varenicline are suggestive, research on the reinforcement-enhancing effects of all cessation medications is likely warranted. Such additional clinical research may provide a more comprehensive understanding of nicotine’s actions on human behavior and suggest how to address loss of these actions during attempts to quit smoking.

## Author Contributions

All listed authors made substantial, direct, and intellectual contributions to this work, and agree to be accountable for all aspects of the research. All have reviewed and approved submission of this article for publication.

## Conflict of Interest Statement

The authors declare that this research was conducted in the absence of commercial or financial relationships that could be construed as potential conflicts of interest.
